# Activity of the prophenoloxidase system and survival of triatomines infected with different *Trypanosoma cruzi* strains under different temperatures: understanding Chagas disease in the face of climate change

**DOI:** 10.1186/s13071-019-3477-9

**Published:** 2019-05-08

**Authors:** Berenice González-Rete, Paz María Salazar-Schettino, Martha I. Bucio-Torres, Alex Córdoba-Aguilar, Margarita Cabrera-Bravo

**Affiliations:** 1 Universidad Nacional Autónoma de México, Ciudad de México, Mexico; 2 Departamento de Microbiología y Parasitología, Facultad de Medicina, Universidad Nacional Autónoma de México, Ciudad de México, Mexico; 3 Departamento de Ecología Evolutiva, Instituto de Ecología, Universidad Nacional Autónoma de México, Apdo. P. 70-275, Circuito Exterior, Ciudad Universitaria, Coyoacán, 04510 Ciudad de México, Mexico

**Keywords:** *Meccus pallidipennis*, *Trypanosoma cruzi*, Strains, Climate change, Temperature, Phenoloxidase activity, Prophenoloxidase activity

## Abstract

**Background:**

Little is known about how human disease vectors will modify their life history patterns and survival capacity as a result of climate change. One case is that of Chagas disease, which has triatomine bugs and *Trypanosoma cruzi* as vectors and parasite, respectively. This work aimed to determine: (i) the activity of the prophenoloxidase system (prophenoloxidase and phenoloxidase activity, two indicators of immune ability) in three intestine regions (anterior midgut, posterior midgutand rectum) of the triatomine bug *Meccus pallidipennis* under three temperature conditions (20 °C, 30 °C and 34 °C) against two *T. cruzi* strains [ITRI/MX/14/CHIL (Chilpancingo) and ITRI/MX/12/MOR (Morelos)], and (ii) whether vector survival varies under these three temperatures after infection by these *T. cruzi* strains.

**Results:**

Our results indicate that prophenoloxidase activity was lower at higher temperatures, that the level of prophenoloxidase activity elicited by each strain was different (higher in Chilpancingo than in Morelos strains), and that prophenoloxidase activity was more intense in the anterior midgut than in the posterior midgut or rectum. Survival rates were lower in insects maintained at higher temperatures and infected by Chilpancingo strains.

**Conclusions:**

These results indicate that climate change could lead to lower prophenoloxidase activity and survival rates in triatomines when infected with different *T. cruzi* strains, which could reduce the vector capacity of *M. pallidipennis*.

## Background

The global temperature increase in recent decades has triggered a multitude of ecological changes, host-parasite interactions being one of such changes [1, 2]. In this regard, understanding the interactions between vector insects and the pathogen agents that they transmit to humans is crucial in assessing the future risk posed by these diseases [3–5]. Recent projections indicate an increase in the distribution of vector insects and the prevalence of the diseases they transmit [6, 7], considering that insects are ectothermic (i.e. basic physiological functions such as locomotion, growth and reproduction are strongly influenced by environmental temperature) [8] and that higher temperatures will promote shorter life-cycles and more rapid reproduction [9, 10]. While this process cannot be generalized for all ectothermic organisms [11], not even for all vectors [9, 12], it is true that empirical data on insects under controlled conditions are much needed to support theoretical models [3, 13].

Triatomines (Hemiptera: Reduviidae) are vectors for *Trypanosoma cruzi* (Kinetoplastida) [14, 15], the causative agent of Chagas disease. In general, the geographical range of triatomines extends from tropical zones, where they withstand temperatures near 40 °C [16, 17], to more temperate zones, with temperatures of about 18 °C [18, 19]. However, the relationship between *T. cruzi* and its vectors could modulate these temperature thresholds [20, 21], possibly due to resource cost-related factors and manipulation by the parasite [22].

*In vitro* studies on triatomines have demonstrated that higher temperatures lead to increased development and reproduction rates for the vector [23, 24], and more frequent feeding events [25–28]. Such an increase would result in a higher risk of *T. cruzi* transmission [28–30], but a balance could be established due to a possible decrease in triatomine survival [26, 31–33]. It is not clear whether this decrease in survival rate is due to an interaction with the parasite, since higher temperatures are expected to favor a more robust prophenoloxidase activity (a proxy of the insect’s immune response) in the vector [28, 34–36].

Triatomine-*T. cruzi* interactions take place in the digestive tract of the vector [37–39]. When triatomines feed from the blood of an infected vertebrate, *T. cruzi* colonize the anterior midgut (AMG), posterior midgut (PMG) and rectum [39, 40].

Interestingly, the AMG is a battlefield between parasites and the host, since almost immediately after blood ingestion, about 80% of parasites die [41, 42]. The prophenoloxidase (proPO) and phenoloxidase (PO) enzymatic cascade is known to be a key element in the immune response associated to the defense against pathogens and their removal [43–46]. In this regard, proPO and PO activity in *M. pallidipennis* were found associated after one week of infection with *T. cruzi* [45].

This study aimed to determine the effect of temperature on the immune response in triatomine-*T. cruzi* interactions, as assessed by proPO and PO activity, in the AMG, PMG and rectum of *M. pallidipennis*. This species is the vector with the highest epidemiological importance in the transmission of Chagas disease in Mexico [47]. It can survive in a temperature range of 18–28 °C when infected by *T. cruzi*, with 27–28 °C being its optimal range [48, 49]. Considering these temperature ranges, triatomine vectors in our study were bred and kept at 20 °C, 30 °C and 34 °C. The reason for choosing the last temperature was two-fold: first, some climate change scenarios predict an increase of 4 °C above the current temperatures [50]; secondly, previous experiments have indicated that *M. pallidipennis* does not survive above 34 °C. As an additional variable, infection with two *T. cruzi* strains was considered in this work. Different isolates and discrete typing units (DTUs) are known to occur in different regions under natural conditions, but it is not clear whether they affect the vector fitness in a differential manner. Increased temperatures are expected to improve triatomine proPO activity [51, 52] at the AMG level, while vector survival rates are expected to decrease [21]. The effect of the different isolates cannot be predicted, but the effects of an isolate type on triatomine survival are expected to remain unaltered under different temperature conditions.

## Methods

### *Meccus pallidipennis* specimens

Newly-moulted, fifth-instar *M. pallidipennis* nymphs were kept in the insectarium of the Laboratorio de Biología de Parásitos (Parasite Biology Laboratory, Department of Microbiology and Parasitology, Faculty of Medicine, Universidad Nacional Autónoma de México) at 30 °C and a relative humidity (RH) of 60% under a 12/12 h light/darkness cycle. These triatomines are descendant of insects collected in the state of Morelos, Mexico, in 1989, with regular introduction of wild specimens. Specimens were randomly selected for the experiments.

### *Trypanosoma cruzi* strains

The strain ITRI/MX/12/MOR (further referred to as Morelos) was obtained from a male *M. pallidipennis* specimen captured and isolated in 2012 in Cuernavaca, Morelos, Mexico. This strain has been characterized as TcI [45]. The strain ITRI/MX/14/CHIL (further referred to as Chilpancingo) was obtained from a female *M. pallidipennis* specimen captured and isolated in 2014 in Chilpancingo, Guerrero, Mexico. This strain has not yet been characterized. Both strains were used to infect female CD-1 mice (15–18 g). Both strains were maintained in CD-1 mice by cyclical passages.

### Infection, incubation and confirmation of infection

#### Infection of M. pallidipennis nymphs

For each strain, 150 nymphs were infected by allowing them to feed from mice that had been inoculated with 20,000 *T. cruzi* metacyclic trypomastigotes/ml 15 days before, to ensure that the parasite was in the exponential stage of growth [45]. Additionally, 150 nymphs were allowed to feed from non-infected female CD-1 mice of the same weight (15–18 g), to be used as a control group. The nymphs of each group were allowed to feed for 15–20 min in the dark, in groups of 5 nymphs per mouse (Morelos, Chilpancingo and control) until they detached themselves from the feeding source and showed clear signs of satiety (the abdomen grew to approximately the double of its pre-feeding size). Based on the levels of parasitemia in the mice, each infected insect ingested approximately 8000 parasites.

#### Temperature challenge

After feeding, the nymphs were placed in plastic jars (one per jar) and labeled for identification. Fifty nymphs infected with the Morelos strain, 50 nymphs infected with the Chilpancingo strain, and 50 control (non-infected) nymphs were incubated at 20 ± 2 °C, 30 ± 2 °C and 34 ± 2 °C and 60% RH for 15 days (acclimatization period) in an incubator (FE-131AD, FELISA, City, Mexico). In total, 150 nymphs were subjected to each temperature.

#### Confirmation of infection

After 15 days of incubation, the rectal content of each specimen was obtained by abdominal compression and examined by direct observation to confirm the presence of *T. cruzi* blood trypomastigotes [45]. A drop of PBS 1× pH 7.2 (Na_2_HPO_4_ 8 × 10^−6^ M, KH_2_PO_4_ 10^−6^ M, KCl 3 × 10^−6^ M, NaCl 10^−4^ M) was placed on a glass slide. A drop of triatomine rectal content was added and the mixture was homogenized [45]. A 10-μl aliquot was taken and microscopically observed under a 40× objective (Olympus CH-2, Center Valley, PA, USA).

#### Extraction of the AMG, PMG and rectum

After the infection was confirmed, the insects were dissected under a stereoscopic microscope (Stemi 2000, Carl Zeiss, Jena, Germany). The legs were removed with dissecting forceps and the insect was placed in a Petri dish at 4 °C. The abdomen was disinfected with 70% alcohol. The connexivum was identified and sectioned to expose the abdominal cavity. Malpighian tubules and the fat body were removed [45]. The digestive system was identified [39], and the AMG, PMG and rectum were dissected. Each region of the triatomine digestive system was separately placed in a 1.5-ml Eppendorf tube with 200 µl of sterile PBS 1× pH 7.2.

#### Processing the AMG, PMG and rectum from infected and non-infected nymphs

The AMG, PMG and rectum from infected and control nymphs were dissected and placed separately in sterile PBS as described above and washed to remove any residue of vertebrate undigested blood. The supernatant was discarded and 200 µl of ice-cold, sterile PBS 1× pH 7.2 were added. The tissue was macerated with a pestle to completely disaggregate it and it was then centrifuged in an Allegra 64R microcentrifuge (Beckman Coulter, Brea, CA, USA) at 9168×*g* for 10 min at 4 °C. Then, 20 µl of supernatant were taken and placed in 180 µl of ice-cold, sterile PBS 1× pH 7.2 (diluted 1:10) [53]. The tubes were kept on ice until used.

#### proPO and PO activity in triatomine AMG, PMG and rectum

proPO and PO activity in triatomine AMG, PMG and rectum were spectrophotometrically determined [43, 54]. Briefly, 25 µl each of AMG, PMG or rectum supernatant from each nymph was placed on a 96-well microplate (Costar 96, Corning, NY, USA). To determine proPO activity, 10 μl of sterile PBS 1× pH 7.2 and 5 µl of bovine α-chymotrypsin (1 mg/ml; Sigma-Aldrich, Saint Louis, MO, USA) were added. The microplate was incubated at 37 °C for 1 h in the dark. Then, 25 µl of L-DOPA (4 mg/ml, Sigma-Aldrich) was added and the plate was incubated at 37 °C for 1 h in the dark [45, 55]. A blank containing 35 µl of sterile PBS 1× pH 7.2 and 5 µl of α-chymotrypsin was included. All samples were analyzed in duplicate.

To determine PO activity, 25 µl each of AMG, PMG and rectum supernatant from each nymph was placed on a 96-well microplate (Costar 96). Ten microliters of sterile PBS 1× pH 7.2 and 25 µl of L-DOPA (4 mg/ml, Sigma) were added. The plate was incubated at 37 °C for 3 h in the dark [45, 56]. A blank containing 5 µl of sterile PBS 1× pH 7.2 and 5 µl of α-chymotrypsin was included. All samples were analyzed in duplicate.

To determine proPO and PO activity, the absorbance of each sample was read in the spectrophotometer at 490 nm every 5 min for 1 h. Enzyme activity was determined by calculating the slope of an absorbance-time plot [45] and using the following equation:

$${\text{Enzyme activity = }}\frac{{m\left( {\frac{Abs}{\hbox{min} }} \right)*vf(L)*F}}{{\varepsilon (M^{ - 1} cm^{ - 1} )*b(cm)}}$$

where m is the slope of the Abs-time (min^−1^) plot, *vf* is the final volume of the reaction (L), *F* is the dilution factor, *ε* is the molar extinction coefficient of dopachrome at 490 nm (3.715 M^−1^ cm^−1^) [54], and *b* is the optical length (0.5 cm).

### Triatomine nymph survival

#### Incubating *M. pallidipennis* nymphs

One-hundred and eighty nymphs (120 infected and 60 non-infected) were incubated as mentioned above. Twenty nymphs infected with the Morelos strain, 20 infected with the Chilpancingo strain and 20 non-infected (controls) were incubated at 20 ± 2 °C. Sixty nymphs were incubated at 30 ± 2 °C, and 60 were incubated at 34 ± 2 °C. Then, all nymphs were monitored daily to determine survival, from infection time to death. An insect was regarded as dead when it failed to move any appendage after being manipulated with dissecting forceps for 1 min.

### Statistical analysis

The Kolmogorov-Smirnoff (K-S) test was used to determine data normality and variance heterogeneity, which indicated a normal distribution of data. Differences in nymph immunocompetent capacity between treatments were assessed by a univariate general linear model, having proPO and PO activity as dependent variables, while infection status (Morelos, Chilpancingo and control), intestine region (AMG, PMG and rectum) and incubation temperature (20 °C, 30 °C and 34 °C) were predictive variables. The significance of the whole model, of each separate predictive variable, and of the interactions between infection status with intestine region and temperature was determined. The latter interaction was studied by comparing groups with 95% confidence intervals. This analysis was performed with the software SPSS v.24.0. All data are expressed as mean enzyme activity ± standard error.

The Mantel-Cox log-rank test was used to determine the effect of infection status (Chilpancingo, Morelos or control) and temperature (20 °C, 30 °C or 34 °C) on nymph survival time. Intergroup differences were determined with the Chi-square test between infected and non-infected groups, and between infected groups. This analysis was performed with the software GraphPad v.7.0.

## Results

### proPO activity with respect to infection status, temperature and intestine region

Significant differences were observed in the linear model with respect to infection status (Chilpancingo, Morelos and control), incubation temperature (20 °C, 30 °C and 34 °C) and intestine region (AMG, PMG and rectum; Table 1). Interactions between all predictive variables were also significant (Table 1).

**Table 1:** Parameters of the general linear univariate model of proPO activity with respect to infection status (infected by Chilpancingo strain, Morelos strain or control), temperature (20 °C, 30 °C and 34 °C), and intestine region (AMG, PMG and rectum), and their interactions

Origin	Type-III SS	*df*	MS	*F*-value	*P-*value
Corrected model	2198.229	26	84.547	237.914	0.0001
Intersection	1034.247	1	1034.247	2910.346	0.0001
Infection status	98.982	2	49.491	139.266	0.0001
Temperature	44.152	2	22.076	62.122	0.0001
Region	1737.878	2	868.939	2445.174	0.0001
Status × temperature	14.070	4	3.518	9.898	0.0001
Status × region	195.991	4	48.998	137.879	0.0001
Temperature × region	79.567	4	19.892	55.975	0.0001
Status × temperature × region	27.589	8	3.449	9.704	0.0001
Error	470.153	1323	0.355		
Total	3702.629	1350			
Corrected total	2668.382	1349			

*Abbreviations*: df, degrees of freedom; MS, mean square; SS, sum of squares

**Fig. 1:**
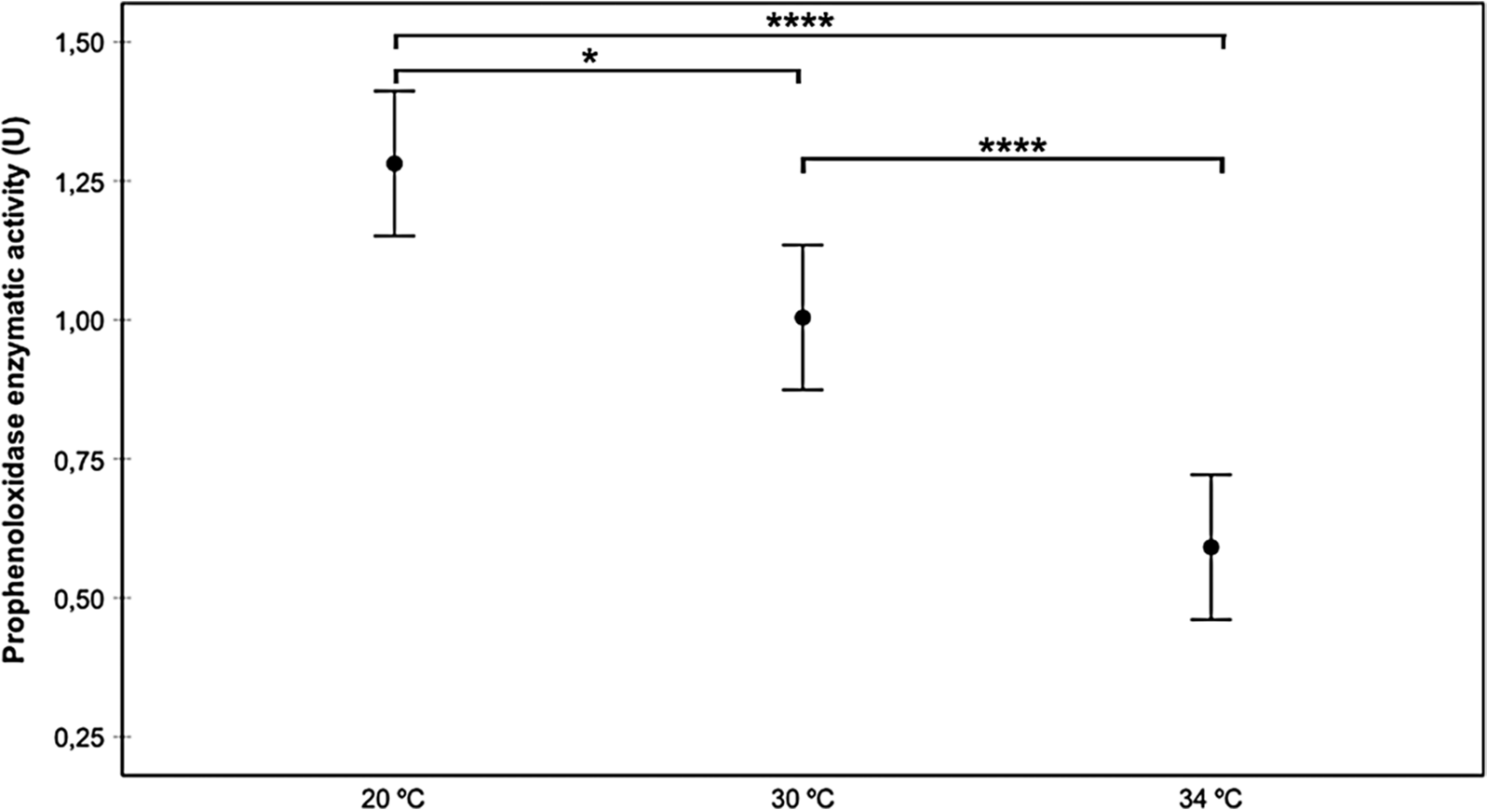
Prophenoloxidase enzyme activity (proPO) in *M. pallidipennis* fifth-instar nymphs kept at three different temperatures (20 °C, 30 °C and 34 °C). *P < 0.05, ****P < 0.0001

**Fig. 2:**
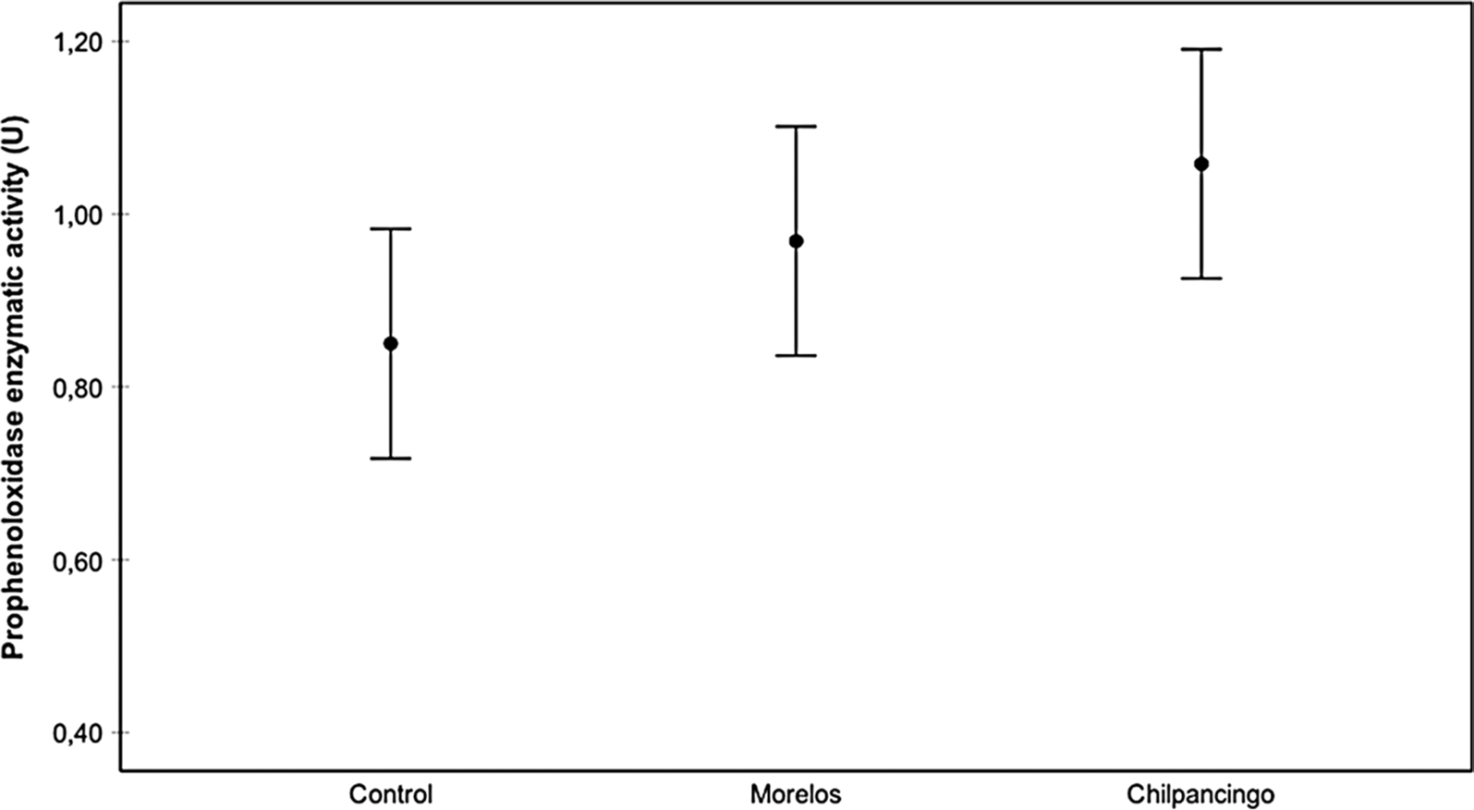
Prophenoloxidase enzyme activity (proPO) in infected (Chilpancingo and Morelos strains) *M. pallidipennis* fifth-instar nymphs and control, non-infected nymphs

**Fig. 3:**
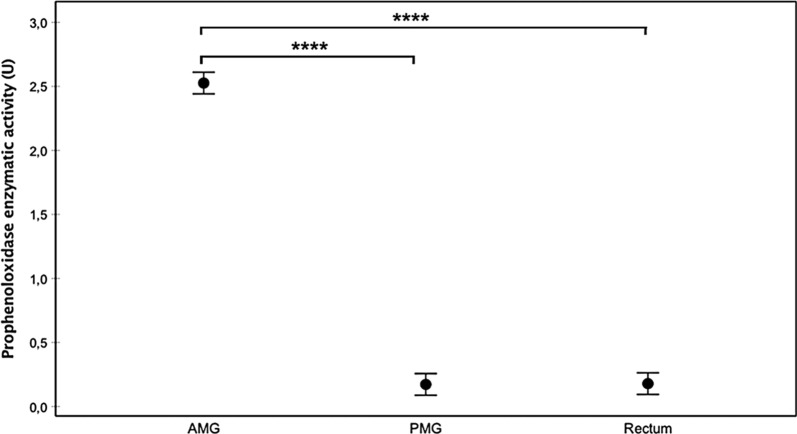
Prophenoloxidase enzyme activity (proPO) in different regions (AMG, PMG and rectum) of the digestive system of *M. pallidipennis* fifth-instar nymphs. ****P < 0.0001

**Fig. 4:**
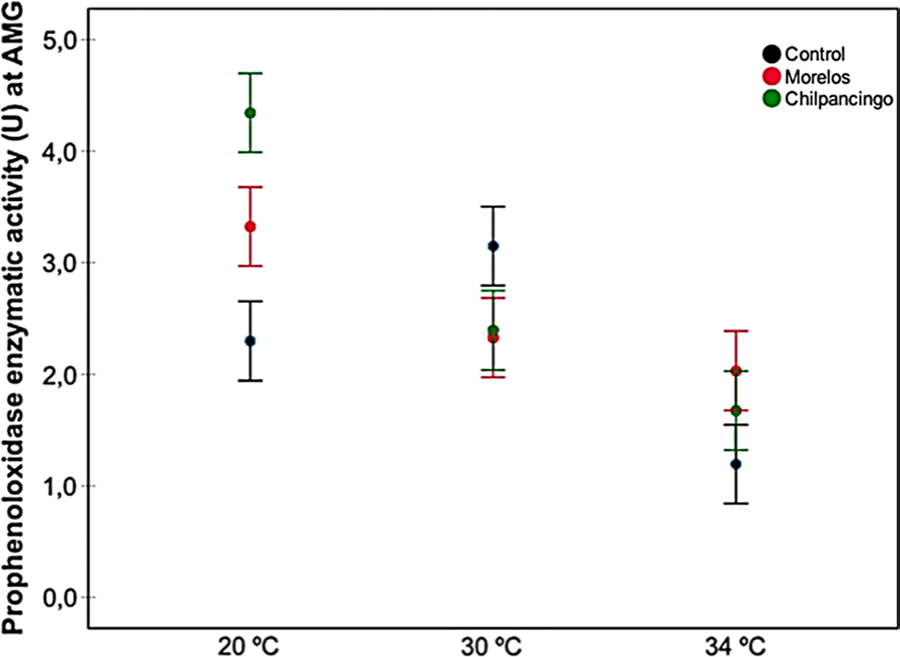
Prophenoloxidase enzyme activity (proPO) in the AMG from infected *M. pallidipennis* fifth-instar nymphs (Chilpancingo and Morelos strains) and control, non-infected nymphs, at different temperatures (20 °C, 30 °C and 34 °C)

In general, as temperature increased, proPO activity decreased (Fig. 1). Infection status also affected proPO activity, with the highest activity rate being observed in the Chilpancingo group, and the lowest in the controls (Fig. 2). The intestine region was also a good predictor of proPO, where the AMG yielded higher activity levels than the PMG and rectum, with no significant differences between the latter groups (Fig. 3). Since the AMG is the site with a significantly higher proPO expression, and no differences were observed between the PMG and rectum, only the AMG will be used in the following comparisons. proPO activity was higher in the Chilpancingo group than in the Morelos and control groups at 20 °C, but these differences were not observed at 30 and 34 °C, which showed a reverse pattern with respect to 20 °C (Fig. 4).

### PO activity with respect to infection status, temperature and intestine region

Significant differences were found with respect to infection status, incubation temperature and intestine region (Table 2). Interactions between all predictive variables were also significant (Table 2).

**Table 2:** Parameters of the general linear univariate model of PO activity with respect to infection status (infected by Chilpancingo strain, Morelos strain or control), temperature (20 °, 30 ° and 34 °C), and intestine region (AMG, PMG and rectum), and their interactions

Origin	Type-III SS	*df*	MS	*F*-value	*P*-value
Corrected model	2022.799	26	77.800	135.060	0.0001
Intersection	1240.545	1	1240.545	2153.567	0.0001
Infection status	9.933	2	4.967	8.622	0.0001
Temperature	108.867	2	54.434	94.496	0.0001
Region	1658.061	2	829.031	1439.184	0.0001
Status × temperature	44.143	4	11.036	19.158	0.0001
Status × region	16.534	4	4.133	7.176	0.0001
Temperature × region	112.613	4	28.153	48.874	0.0001
Status × temperature × region	72.647	8	9.081	15.764	0.0001
Error	762.104	1323	0.576		
Total	4025.448	1350			
Corrected total	2784.903	1349			

*Abbreviations*: df, degrees of freedom; MS, mean square; SS, sum of squares

**Fig. 5:**
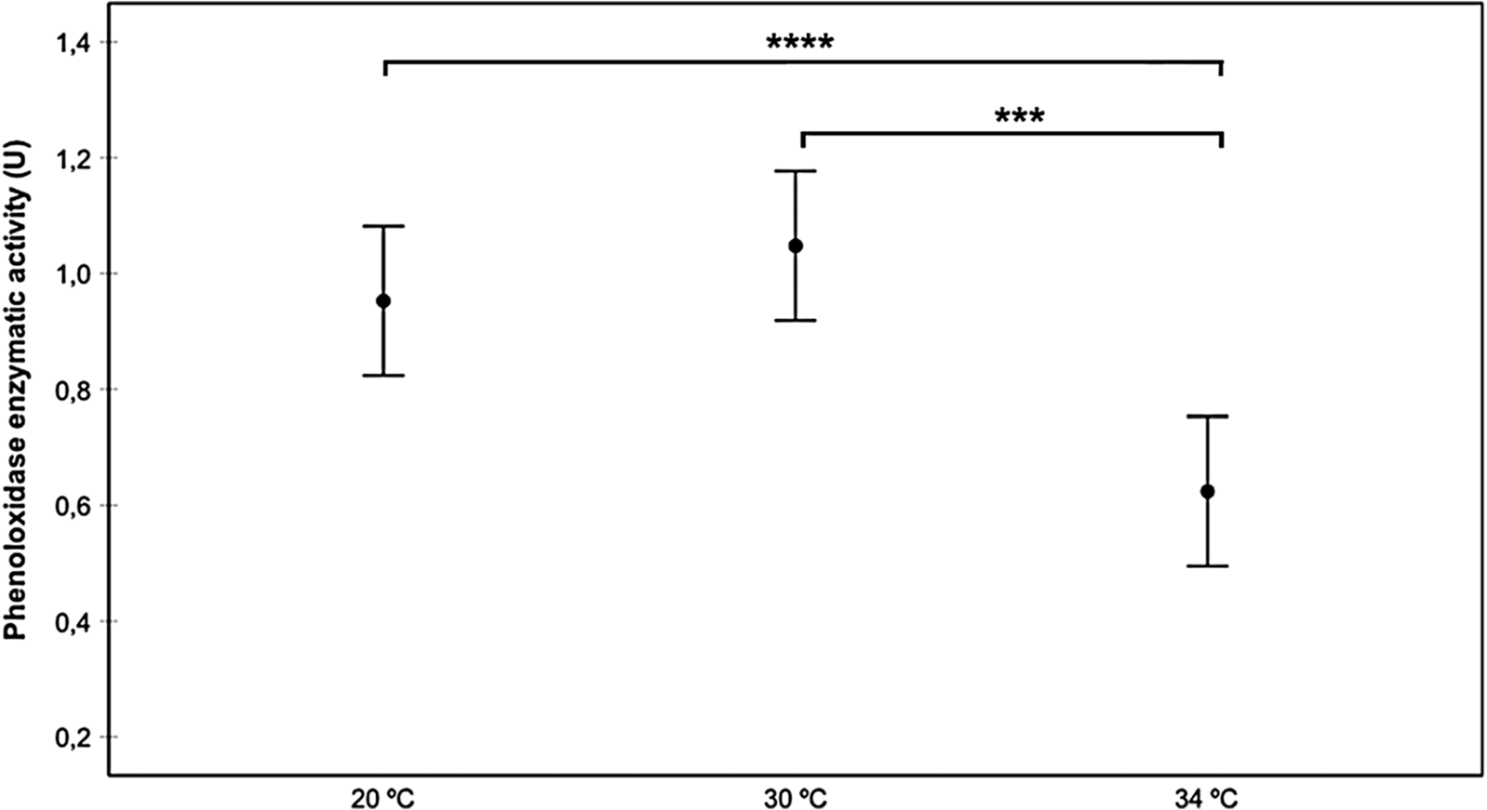
Phenoloxidase enzyme activity (PO) in *M. pallidipennis* fifth-instar nymphs kept at three different temperatures (20 °C, 30 °C and 34 °C). ***P < 0.0005, ****P < 0.0001

**Fig. 6:**
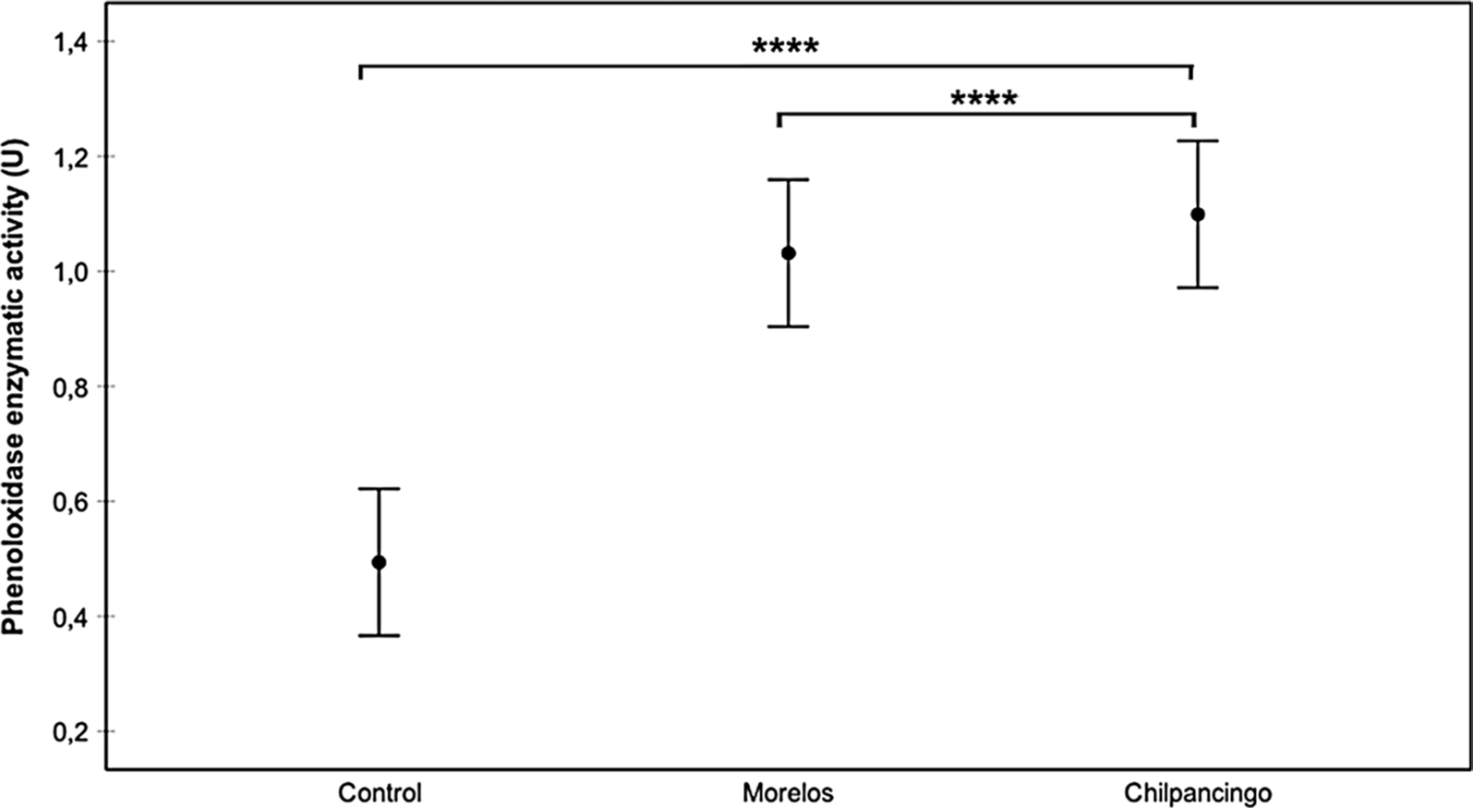
Phenoloxidase enzyme activity (PO) in infected *M. pallidipennis* fifth-instar nymphs and control, non-infected nymphs. ****P < 0.0001

**Fig. 7:**
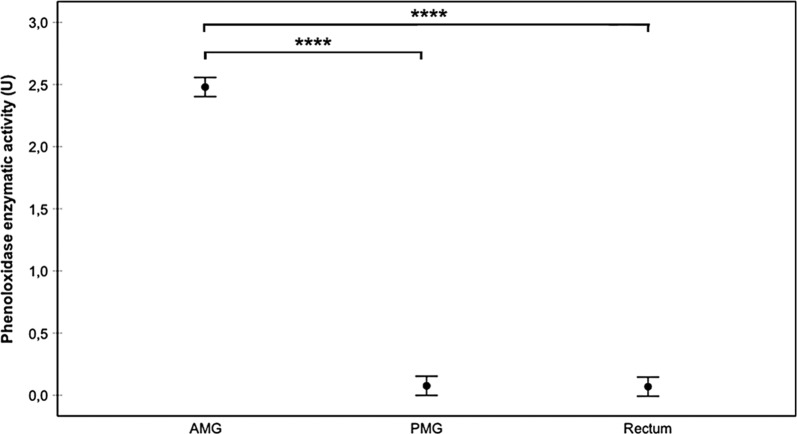
Phenoloxidase enzyme activity (PO) in different regions (AMG, PMG and rectum) of the digestive system of *M. pallidipennis* fifth-instar nymphs. ****P < 0.0001

**Fig. 8:**
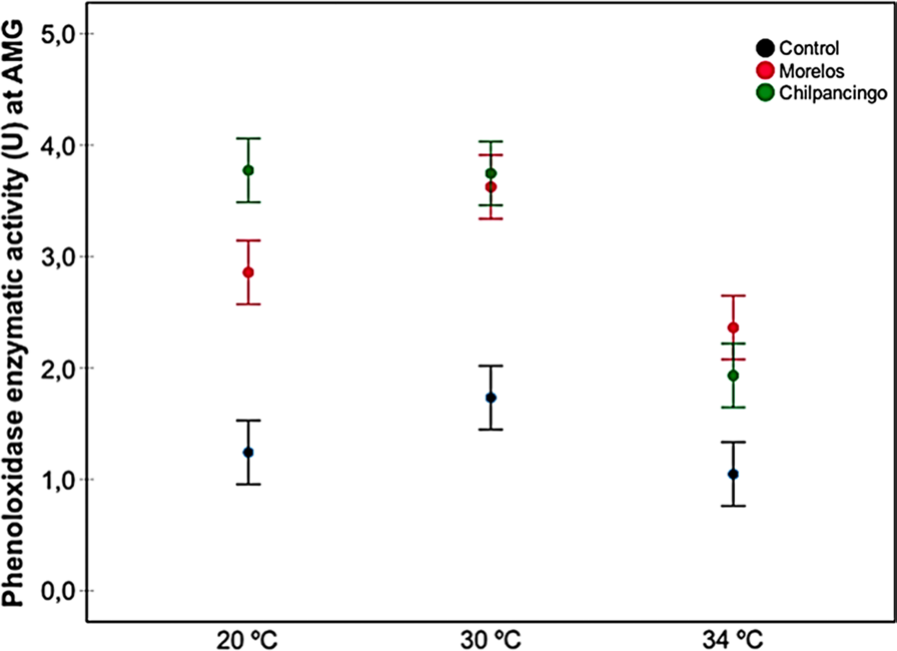
Phenoloxidase enzyme activity (PO) in the AMG from infected *M. pallidipennis* fifth-instar nymphs and control, non-infected nymphs, at different temperatures (20 °C, 30 °C and 34 °C). Note that infected nymphs showed a higher activity than control specimens, irrespective of the temperature they were exposed to

The highest PO activity values were observed at 30 °C and were significantly lower at 34 °C (Fig. 5). With respect to infection status, the Chilpancingo group showed the highest PO activity, and control showed the lowest (Fig. 6). With respect to intestine region, the AMG yielded the highest response, with no differences between the PMG and rectum (Fig. 7). As with proPO, PO activity levels in the AMG will be used in the following comparisons, since no differences between the PMG and rectum groups were observed. PO activity was higher in the Chilpancingo group than in the Morelos and control groups at 20 °C, but these differences were not observed at 30 or 34° C (Fig. 8). Note that while the Chilpancingo group showed higher PO activity than the Morelos group at 20 °C, this pattern was opposite at 30 and 34 °C.

### Effect of temperature on survival of fifth-instar *M. pallidipennis* nymphs

**Fig. 9:**
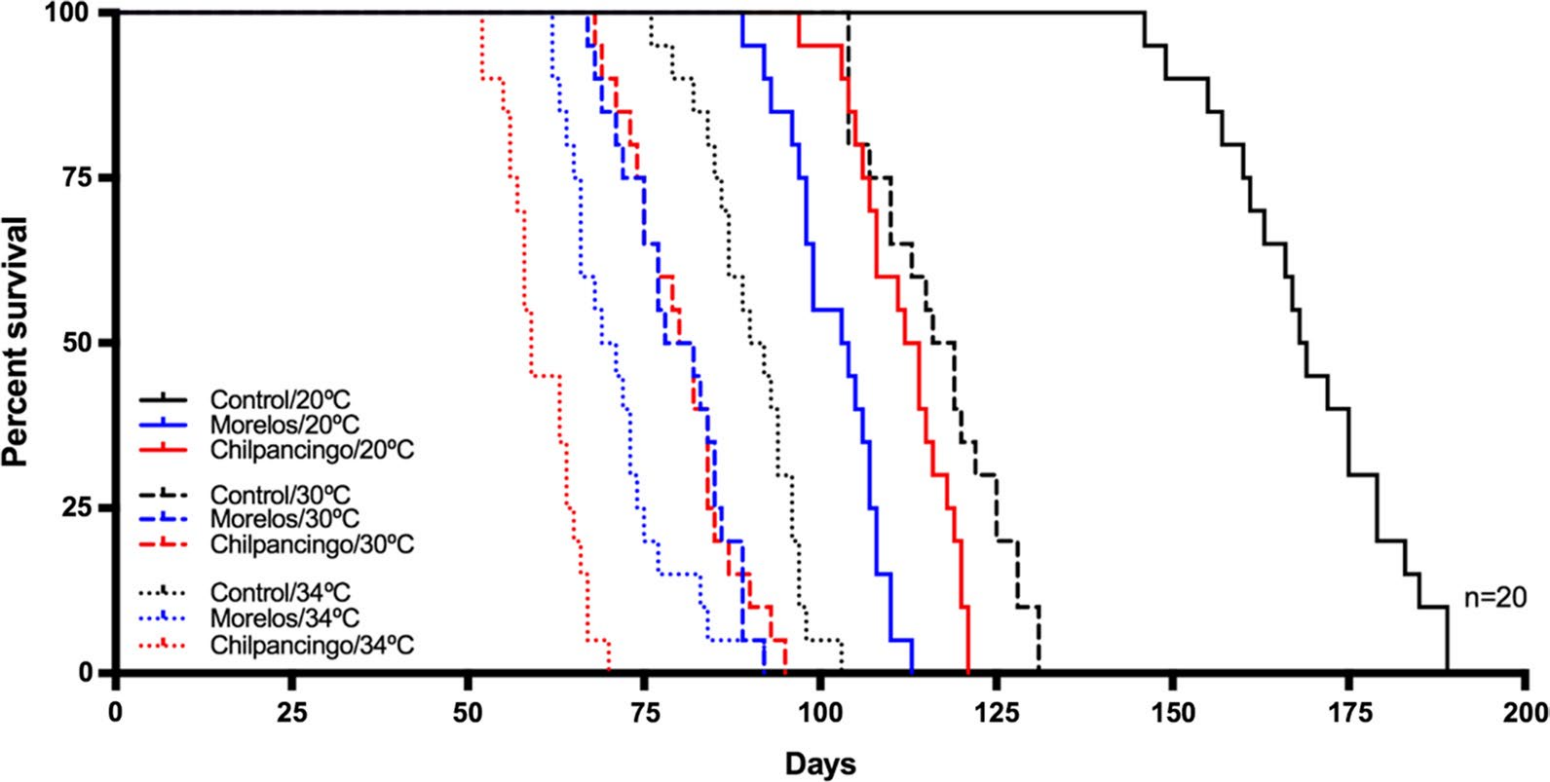
Survival in infected *M. pallidipennis* fifth-instar nymphs and control, non-infected nymphs, at different temperatures (20 °C, 30 °C and 34 °C)

**Fig. 10:**
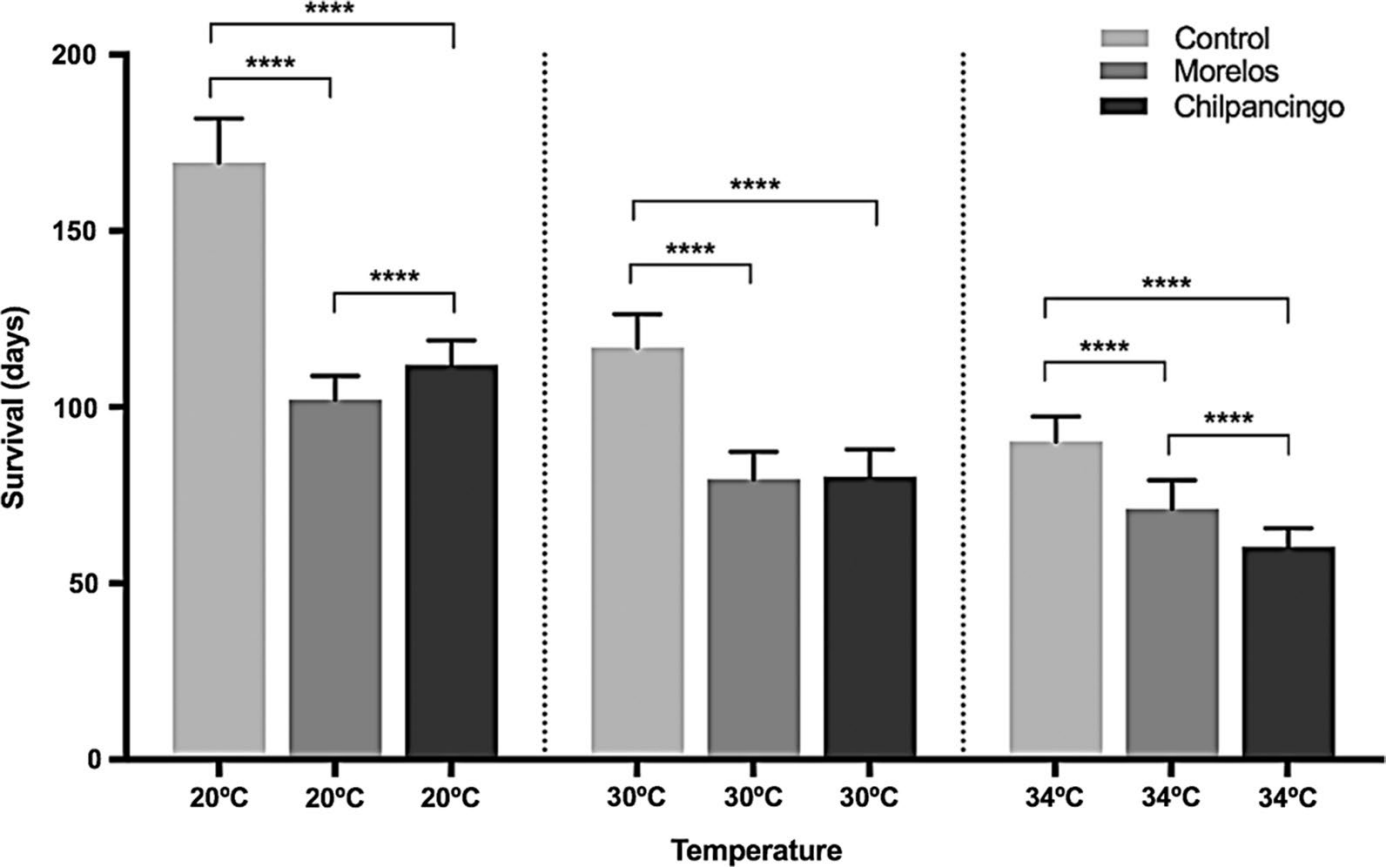
Survival time in *M. pallidipennis* fifth-instar nymphs at different temperatures (20 °C, 30 °C and 34 °C). ****P < 0.0001

In general, increased temperatures reduced triatomine lifespan (Fig. 9; Table 3). Infection status had some effect as well, decreasing insect survival. The Morelos strain had a higher effect on nymph survival than the Chilpancingo strain at 20 °C, but this effect was inverted at 34 °C (Figs. 9, 10).

**Table 3:** Survival of *M. pallidipennis* nymphs with respect to infection status (Chilpancingo strain, Morelos or control) and maintained at different temperatures

Temperature (°C)	Status	Sample size (*n*)	Mean survival (days)
20	Control	20	166
	Morelos	20	102
	Chilpancingo	20	112
30	Control	20	117
	Morelos	20	80
	Chilpancingo	20	80
34	Control	20	90
	Morelos	20	71
	Chilpancingo	20	60

## Discussion

The immune capacity of triatomines against infection by *T. cruzi* was enhanced when the temperature was increased from 20 °C to 30 °C, but this pattern changed dramatically at 34 °C. Our results, indicating a more efficient prophenoloxidase activity before a subtle increase in temperatures, are in agreement with reports on other insects [57]. This could be explained as the result of a better prophenoloxidase activity *via* phenotypic plasticity at moderately higher temperatures, which become inviable as temperature reaches a lethal threshold. In terms of defense against a pathogen, a temperature close to lethal levels could preclude the action of the triatomine prophenoloxidase activity against an infection, which is consistent with our results on nymph survival. Conversely, the insect may respond in the form of fever during infection, which may help them to fight off *T. cruzi* [58]. If this were the case, however, one would expect that a high temperature may help insects to produce such an antiparasitic effect, but it is not the case as survival was lower as temperature rose. It is worth noting, however, that despite the strong effect of higher temperatures on reduced survival, this does not mean that the triatomine bugs do not rely on a fever response to deal with pathogens. Actually, other studies have found that triatomines show fever responses when sick [34]. It would be interesting to see whether *M. pallidipennis* bugs increase their fitness when allowed to choose ambient temperatures while facing different *T. cruzi* isolates.

The prophenoloxidase activity against *T. cruzi* in triatomines is known to be closely related with the intestinal tract [45]. Since the AMG is the anatomic region first colonized by the parasite and where its reproduction in the vector begins (and to a lesser extent in the PMG [41, 42]), the prophenoloxidase activity in this region is expected to be more robust, as our results indicate. This is consistent with the massive death of the parasite observed within a few hours of infection [42], although it is not clear why the prophenoloxidase activity is not high enough in other gut regions to complete parasite removal. A possible explanation is that the cost of infection can affect AMG only, where parasite replication and resource sequestering could be considerably higher. Alternatively, it could be energetically prohibitive for the triatomine to maintain a high prophenoloxidase activity to kill the few surviving parasites. Such a tolerance would imply that relatively low parasite levels would be more preferable for the insect than complete removal [59, 60].

In contrast with the prophenoloxidase activity, our expectations with respect to survival were fulfilled. The negative effects of the parasite on triatomine fitness are not new. For instance, Botto-Mahan [61] found that *T. cruzi* infection delays the development and reduces survival rates in the triatomine *Mepraia spinolai*. Studies more closely related to our work found that *T. cruzi* reduced the survival of the triatomine *Rhodnius prolixus*, but only at 20 °C and 30 °C [31, 34]. In our case, the insects were more affected by the Chilpancingo strain than by the Morelos strain. These effects on survival are related to those on the prophenoloxidase activity, suggesting that the vector allocates more resources to defend itself from a more dangerous pathogen. The more rapid death in insects infected with the Chilpancingo strain could be because this strain extracted more resources from the insect than the Morelos strain, but the precise mechanisms underlying infection costs in triatomines are still unknown [21]. Interestingly, the lethal effects of the Chilpancingo strain are even more perceptible at higher temperatures (34 °C). On one hand, these results do not agree with the report by Elliot et al. [31], who did not find a negative effect of high temperatures on *R. prolixus* survival. However, much lower temperatures (30 °C) were used in those experiments than in our work (34 °C). It is possible that our temperature was closer to the lethal threshold for *M. pallidipennis* than the one used by Elliot et al. [31] for *R. prolixus*. On the other hand, our results may help us to understand the adaptation of triatomines to thermal thresholds in nature. For instance, it has been reported that the optimal temperature is 30 °C and the upper thermal limit for *M. pallidipennis* rarely seems to exceed 34 °C [48, 49], which is in agreement with our results. While temperature levels in our study were selected based on the thresholds predicted by different climate change scenarios, it is likely that these scenarios are already occurring in some torrid areas of Mexico inhabited by *M. pallidipennis* [62].

Finally, our results have implications for our understanding of the dynamics of Chagas disease in nature. Assuming our experimental scenarios as realistic, the risk of Chagas disease in terms of vector effectivity, measured as triatomine lifespan, would decrease with rising global temperatures. Clearly, this conclusion disregards other complex factors, like the possibility that triatomines modify their feeding behavior as a result of the infection. In this regard, previous studies have reported that infected triatomines feed and defecate more frequently [22] and are more active. Thus, while it can be assumed that infected triatomines live shorter lives, the risk they pose may be unchanged if their feeding rate is higher than that of non-infected insects. If feeding rate is higher, climate change could make vector triatomines as dangerous as today, or even more. Future experiments should determine whether higher temperatures modify the vector capacity of triatomines.

## Conclusions

Our experimental results indicate a covariation between triatomine immune ability and temperature using two *T. cruzi* strains: (i) the prophenoloxidase activity was less robust at temperatures that simulate climate change, and (ii) prophenoloxidase activity was higher in the Chilpancingo than in Morelos strains. Furthermore, triatomine survival was reduced at high temperatures that simulate global change which was more drastic when infected by the Chilpancingo strains. These results imply that climate change scenarios can reduce both triatomine fitness and vector capacity.

## Abbreviations

AMG: anterior midgut
PMG: posterior midgut
proPO: prophenoloxidase
PO: phenoloxidase
DTU: discrete typing unit
U: enzymatic unit
